# Cognitive tasks and cerebral blood flow through anterior cerebral arteries: a study via functional transcranial Doppler ultrasound recordings

**DOI:** 10.1186/s12880-016-0125-0

**Published:** 2016-03-12

**Authors:** Héloïse Bleton, Subashan Perera, Ervin Sejdić

**Affiliations:** Department of Electrical and Computer Engineering, University of Pittsburgh, Pittsburgh, PA USA; Division of Geriatric Medicine, University of Pittsburgh, Pittsburgh, PA USA

**Keywords:** Anterior cerebral arteries, Cerebral blood flow, Functional transcanial Doppler ultrasound, Signal processing

## Abstract

**Background:**

Functional transcanial Doppler ultrasound (fTCD) is a convenient approach to examine cerebral blood flow velocity (CBFV) in major cerebral arteries.

**Methods:**

In this study, the anterior cerebral artery (ACA) was insonated on both sides, that is, right ACA (R-ACA) and left ACA (L-ACA). The envelope signals (the maximum velocity) and the raw signals were analyzed during cognitive processes, i.e. word-generation tasks, geometric tasks and resting state periods separating each task. Data which were collected from 20 healthy participants were used to investigate the changes and the hemispheric functioning while performing cognitive tasks. Signal characteristics were analyzed in time domain, frequency domain and time-frequency domain.

**Results:**

Significant results have been obtained through the use of both classic/modern methods (i.e. envelope/raw, time and frequency/information-theoretic and time-frequency domains). The frequency features extracted from the raw signals highlighted sex effects on cerebral blood flow which revealed distinct brain response during each process and during resting periods. In the time-frequency analysis, the distribution of wavelet energies on the envelope signals moved around the low frequencies during mental processes and did not experience any lateralization during cognitive tasks.

**Conclusions:**

Even if no lateralization effects were noticed during resting-state, verbal and geometric tasks, understanding CBFV in ACA during cognitive tasks could complement information extracted from cerebral blood flow in middle cerebral arteries during similar cognitive tasks (i.e. sex effects).

## Background

Distribution patterns of cerebral blood flow can be described by neuroimaging techniques such as functional magnetic resonance imaging, single photon emission computed tomography, positron emission tomography or the xenon-clearance technique. All these methods have a high spatial resolution [1–4]. Despite their advantages, these methods restrict patient movements and usually have a low temporal resolution. The hemodynamic features of major cerebral arteries and their rapid variations in normal and pathological conditions can be characterized by functional transcanial Doppler ultrasound. Functional transcanial Doppler ultrasound (fTCD) is a non-invasive blood velocity measurement approach [5]. This technique uses the fact that cerebral perfusion is linked to neural activation which is translated into cerebral perfusion changes during cognitive tasks [6, 7]. It has a high temporal resolution due to continuous insonation of cerebral blood flow velocity. The velocity measurement is closely linked to cerebral blood flow in the event that the diameter of cerebral arteries does not change during the insonation. Multiple studies showed that perfusion area and diameter of cerebral arteries do not change during mental processes [8–10]. Thus, blood flow velocity evolutions are due to modifications in cerebral metabolism because of cerebral activities.

fTCD has been studied during cognitive or physical tasks for both healthy participants and patients affected by neurological disorders (e.g., stroke, autism, epilepsy) [11–15]. Previous publications have examined the effects of visual perception [16, 17], auditory perception [18, 19], language processes [20, 21], spatial processes [22, 23], memory processes [24], other cognitive/mental tasks and other neurological disorders [13, 25, 26] on cerebral blood flow using fTCD. These publications pointed that the main advantages of a fTCD system include its price, easiness-to-use and its minimally stressful character [27].

fTCD is mainly used to target major cerebral arteries [28]. Usually, transducers are placed on the thinnest parts of head bone which are the acoustic windows of the skull allowing to monitor activities on cerebral arteries of the circle of Willis. The transtemporal window enables us to reach the middle (MCA), anterior (ACA) and posterior (PCA) cerebral arteries. The transforaminal window enables us to reach the basilar and the vertebral arteries; while the transorbital window reaches the ophthalmic and the internal arteries [29]. Arteries are identified by understanding the depth of insonation, the transducers position and the flow direction [30]. The most commonly insonated arteries are the ACA, the MCA and the PCA [31]. Each of these arteries supplies blood to different areas: the ACA supply to the medial regions, the MCA supply to the lateral regions and the PCA supply to the posterior basomedial regions. The MCA is most usually insonated in studies about cognitive processes [20, 32–34], as 80 % of blood to the brain is delivered by the MCA. The ACA could be also insonated during high cognitive functions such as arithmetic problems or receptive language [35–37]. As ACA are deeper than MCA [29], insonating ACA could provide complimentary information to signals acquired from MCA in order to gain further understanding of cerebral blood flow characteristics while performing mental activities [30, 38, 39].

Previous publications regarding cerebral blood flow velocities during mental stimuli on MCA highlighted the left and right hemispheric dominance introduced during the geometric task and the word-generation task respectively. However, the brain blood flow in ACA is closely linked to the activity in MCA [32, 33, 35]. In fact, lateralization in the ACA blood flow was predicted while performing cognitive processes (i.e. evolutions of cerebral blood flow velocity can be explained by the changes in the MCA during mental challenges). Moreover, handedness and sex appeared to have effects on brain response during activation periods. Distinct functioning hemispheric dominance may be foreseen according to sex and handedness [40].

Our hypothesis was that cognitive tasks affect the cerebral blood flow velocities in ACAs similarly to those changes observed in cerebral blood flow velocities in MCAs. To examine our hypothesis, we collected both raw signals and maximal velocity signals (usually called the spectral envelope signals [41]). A few fTCD studies only examined envelope signals and may lack information contained in raw signals [5, 31, 42]. Previous studies highlighted the significance of data embodied in raw signals during resting periods [43, 44]. Envelope signals are usually extracted from raw signals, which are a sum of signals corresponding to erythrocytes movement at different velocities. Raw results which are used to calculate envelope signals, may contain exhaustive information about the activation stimuli and resting-state periods.

Our major contributions include the understanding of signal patterns in various domains (time, frequency, and time-frequency) for raw and maximum velocity signals. Features from the classical analysis were examined (i.e. time and frequency approaches). We also used modern analysis characteristics from information-theoretic and time-frequency domains which have not been examined in previous studies about brain response during mental tasks [45]. Additionally, the current study complements the study of the ACA resting-state characteristics [43] and follows outcomes from MCA results during resting periods and activation stimuli (word-generation and geometric rotation tasks) [45]. These two previous papers employed the same methodology. The repercussions of sex and handedness on CBFV were also examined.

## Methods

### Subjects

Twenty able-bodied participants have taken part in the experiment (Males/Females = 9/11, 22.1±1.86 years old; 171±10.1 cm; 68.9±27.3 kg). Table 1 summarizes participant demographic details. No participant had a history of heart murmurs, strokes, concussions, migraines or other brain-related injuries or neurological diseases. At first, the subjects were asked to sign the consent form approved by the University of Pittsburgh Institutional Review Board. The entire study was approved by the University of Pittsburgh Institutional Review Board.

**Table 1:** Demographic information

Distribution	Male	Female	All
Age (years old)	22.3 ± 1.64	22.0 ±2.00	22.1 ±1.86
Height (cm)	180 ±7.26	163 ±5.39	171 ±10.1
Weight (kg)	91.6 ±29.3	52.6 ±5.89	68.9 ±27.3

The handedness of each participant was tested using the Edinburgh Handedness Inventory test [46]. This technique is one of the most widely used method for measuring both the direction and the degree of handedness [47, 48]. Subjects had to choose their hand preferences based on a list of activities. They could assign 1 or 2 for each activity (1 for a weak preference or 2 for a strict preference between right or left hand). The result was scored based on the formula:

(1) $$ Score=\frac{\sum{X_{i}(R)}-\sum{X_{i}(L)}}{\sum{X_{i}(R)}+\sum{X_{i}(L)}}  $$

where *X*_*i*_(*R*) or *X*_*i*_(*L*) can take values of 0, 1 or 2 according to the domination of right/left domination. Positive score leads to right-handedness whereas negative score leads to left-handedness. This study was restricted to the analysis of handedness direction. In fact, a majority of previous publications about fTCD and brain cognitive response focused on the effects of handedness direction [35, 49, 50]. 16 subjects were right-handed (mean score of 64), 3 subjects were left-handed (mean score of -63) and one was ambidextrous. Table 2 summarizes the Edinburgh Handedness test results.

**Table 2:** Handedness information

Distribution	Right-Handed	Left-Handed	Ambidextrous
Sex	8 males, 8 females	1 male, 2 females	1 female
Average score	64	-63	0

### Procedure

ACA cerebral blood flow was assessed thanks to a SONARA TCD System (Carefusion, San Diego, CA, USA). Two 2 MHz transducers were placed on the left side and the right side of the skull on transtemporal windows to acquire bilateral cerebral blood flow measurements. The temporal windows are found above the zygomatic arch [38]. Transducers were fixed with a headset (5 cm in front of the ears) and were positioned to reach ACA. Additionally, the end-tidal carbon dioxide *ETCO*_2_ (BCI Capnocheck Sleep Capnograph, Smiths Medical, Waukesha, Wisconsin, USA) was monitored along with respiration rate, electrocardiogram, head movement and skin conductance via a multisystem physiological data monitoring system (Nexus-X, Mindmedia, Netherlands). The *ETCO*_2_ levels may have repercussions on cerebal blood flow in the ACA.

**Fig. 1:**
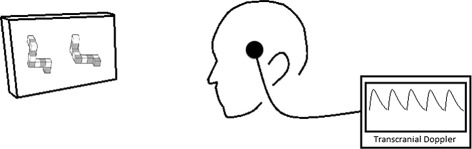
Setup for the fTCD study

**Fig. 2:**
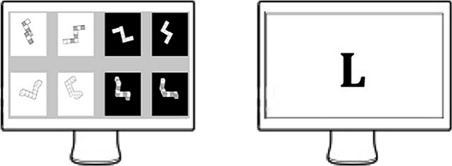
A sample of the on-screen geometric and word-generation stimuli

The participants were asked to complete two 15-minute cognitive parts interspersed by a 5-minute break. However, we did not collect data during these five minutes. Each 15-minute part comprises 5 mental rotation tasks, 5 word generation tasks and 5 resting conditions between each cognitive task. Each of these lasted for 45 seconds. The order of cognitive tasks was randomly assigned, but it was counterbalanced. Figures 1 and 2 illustrate the fTCD setup.

After the acquisition of R-ACA and L-ACA cerebral blood flow data in the form of audio file, information are extracted from audio files sampled at 44100 Hz. Raw data were downsampled to 8820 Hz to speed up data processing.

#### Resting-state

During 5-minutes breaks and during the resting-state, participants were requested to remain awake, maintain a thought-free mental state and keep quiet.

#### Geometric rotation task

During the 45-seconds mental rotation tasks, pairs of images were randomly selected from a database constructed from 3-D cubes [51]. Pairs were displayed for 9 seconds each. Participants were asked to rotate shapes to find which ones are identical or mirror symmetrical.

#### Word generation task

Participants generated words silently based on letters randomly chosen and displayed at the beginning of each period. Subjects were cautioned not to vocalize any words in order to avoid any brain activations related to speech regions [52].

## Feature extraction

Three common parameters in statistics were considered: standard deviation, skewness, kurtosis of the signal amplitude [53]. Statistical parameters from the envelope and the raw signals were extracted. Standard deviation of a signal estimates the spread of a distribution [53, 54]. The skewness of the amplitude distribution quantifies the asymmetry of the distribution [53, 55]. The kurtosis of a distribution evaluates the behavior of the distribution close to the boundaries [53]. These statistical features characterize signals from the right side and from the left side of ACA.

The cross-correlation coefficient at the zero lag between the L-ACA signal and the R-ACA signal was used to demonstrate whether L-ACA signal and R-ACA signal are related:

(2) $$ {CC}_{X_{R-ACA}/Y_{L-ACA}}=\frac{1}{N}\sum\limits_{i=1}^{N} (x_{i} y_{i})  $$

where the signal *X*_*R*−*ACA*_={*x*_*i*_} and *Y*_*L*−*ACA*_={*y*_*i*_}, *i*=1,⋯,*N* are extracted from the right and the left side of the ACA.

### Information-theoretic feature

Information-theoretic features were also taken into consideration. The Lempel-Ziv complexity (LZC) and the entropy rate were extracted. These measures provide information about the complexity and the regularity of signals.

The amount of new patterns formation through finite time sequences is determined by the Lempel-Ziv complexity [56]. In fact, it estimates the predictability and the randomness of the signal [57, 58]. The Lempel-Ziv measure is often used in applications of analysis of biomedical signals [56, 59]. First, the signal amplitude is converted into a finite binary series. It is divided into 100 finite spaces defined thanks to 99 thresholds, *T*_*h*_, 1≤*h*≤99, *h*∈ZZ^+^. The threshold is usually chosen as the median of the signal [60]. Secondly, the quantized signal ${X_{1}^{n}}=\{x_{1},x_{2},\cdots,x_{n}\}$ is divided into blocks. Each block is series of successive data of length *L*. All block can be defined as the following formula [61]:

(3) $$ B={X_{j}^{l}}=\{x_{j},x_{j+1},\cdots,x_{l}\}, 1\le j \le l \le n, j,l \in Z^{+}  $$

where the length *L* of the block is defined by *j*−*l*+1. For each L, every block is tested from left to right. A counter *c* is defined and it increases by one unit if a block has not already appeared in previous *j* and *l*. Finally, the LZC is given as the following formula:

(4) $$ LZC=\frac{c({log}_{100}c+1)}{n}  $$

where *c* denotes the final value of the counter at the end of the signal analysis and n represents the total of quantized levels in the signal.

The entropy rate *ρ* quantifies the regularity in a distribution [62]. First, the signal needs to be normalized to zero mean and unit variance (subtracting *μ*_*X*_ and dividing by *σ*_*X*_) and quantized into 10 equal levels. The quantized signal *X*={*x*_1_,*x*_2_,⋯,*x*_*n*_} is decomposed and grouped into blocks of length L, 10≤*L*≤30, which are finite series of consecutive points in the quantized signal such as *Ω*_*L*_={*ω*_1_,*ω*_2_,⋯,*ω*_*n*−*L*+1_} [63].

(5) $$ \omega_{i}=10^{L-1}x_{i+L-1}+10^{L-2}x_{i+L-2}+\cdots+10^{0}x_{i}  $$

where L is the length of successive series and *ω*_*i*_ is classified between 0 and 10^*L*^−1. The Shannon entropy *S*(*L*) of *ω*_*L*_ given the quantized signal *Ω*_*L*_, where X takes discrete values *ω*_*j*_ with probability *p*_*j*_ is defined as [64]:

(6) $$ S(L)=\sum\limits_{j=0}^{10^{L-1}} p_{j} ln p_{j}  $$

where *p*_*j*_ is the approximated sample joint probability of the pattern *j* in *Ω*_*L*_ with the understanding that $\sum _{j=1}^{n-L+1}p_{j}=1$ with 0≤*p*_*j*_≤1*i*=1,⋯,*n*−*L*+1. The normalized entropy rate is computed as the following formula [65, 66]:

(7) $$ N(L)=\frac{S(L)-S(L-1)+S(1)pe(L)}{S(1)}  $$

where *pe*(*L*) is the percentage of the integers in the L-dimensional phase space that appeared only once and where *S*(1)*pe*(*L*) is added due to the limited number of samples and the underestimation of *S*(*L*)−*S*(*L*−1) for larger L. Given that the first term decreases while the second term increases with L, the goal of this method is looking for the minimum of the previous function. This minimum is an index of complexity. Finally, the regularity index *ρ* of the signal is defined by the following relation [66]:

(8) $$ \rho=1-min(N(L))  $$

where *ρ* is ranged from 0 which is equivalent to a maximal randomness to 1 which corresponds to a minimal regularity.

The cross-entropy rate quantifies the coupling of the entropy rate between two stochastic processes. It predicts data in a signal from previous and current information in another signal. Instead of making one signal normalized, both *X* and *Y* were processed (normalized, quantized and computed according to the previous method), yielding ${\Omega _{L}^{X}}$ and ${\Omega _{L}^{Y}}$. Finally, the cross-entropy rate$\Omega _{L}^{X|Y}$ which represents the information rate available in one of the samples of the quantized signal x when a pattern of *L*−1 samples of the quantized signal y is established was constructed as [65]:

(9) $$ \omega_{i}^{X|Y}=10^{L-1}x_{i+L-1}+10^{L-2}y_{i+L-2}+\cdots+10^{0}y_{i}  $$

The normalized cross-entropy NC of *X*|*Y* is figured out as:

(10) $$ {NC}_{X|Y}(L)=\frac{S_{X|Y}(L)-S_{Y}(L-1)+S_{X}(1){pe}_{X|Y}(L)}{S_{X}(1)}  $$

where *S*_*X*_(*L*), *S*_*Y*_(*L*) and *S*_*X*|*Y*_ represent the Shannon entropies of ${\Omega _{L}^{X}}$, ${\Omega _{L}^{Y}}$and $\Omega _{L}^{X|Y}$. *pe*_*X*|*Y*_(*L*) is the rate of data in $\Omega _{L}^{X|Y}$ that appeared only once and *S*_*X*_(1)*pe*_*X*|*Y*_(*L*) is added due to the limited number of samples and the underestimation of *S*_*X*|*Y*_(*L*)−*S*_*Y*_(*L*−1) for larger L. As the previous method, the goal is looking for the minimum of the previous function. The index of synchronization was used as the cross-entropy rate characteristic:

(11) $$ \Lambda_{X|Y}=1-min({NC}_{X|Y}(L), {NC}_{Y|X}(L))  $$

where *Λ*_*X*|*Y*_ is between 0 which denotes that X and Y are independent processes and 1 which proves a synchronization of X and Y.

### Frequency analysis

Spectral changes of the recorded signals were examined through the peak frequency, the centroid frequency, the bandwidth of the spectrum [67, 68]. The peak frequency is associated with the maximal spectral power:

(12) $$ f_{p}={arg}_{f}max\{|F_{X}(f)|^{2}\}  $$

where *F*_*X*_(*f*) is the Fourier transform of the signal *X* and *f*_*max*_ in this study was 8820 Hz. The spectral centroid is defined as the center of gravity of the spectrum [69]:

(13) $$ f_{c}=\frac{\int_{0}^{f_{max}} f|F_{X}(f)|^{2}\, \mathrm{d}\,f}{\int_{0}^{f_{max}}|F_{X}(f)|^{2}\, \mathrm{d}\,f}  $$

The bandwidth of the spectrum which represents the difference between the higher and lower frequencies of the spectrum measures the spreadness of the frequency components:

(14) $$ B=\sqrt{\frac{\int_{0}^{f_{max}} (f-\widehat{f})^{2}|F_{X}(f)|^{2}\, \mathrm{d}\,f }{\int_{0}^{f_{max}} |F_{X}(f)|^{2}\, \mathrm{d}\,f }}  $$

The bandwidth represents the squared differences between the spectral centroid and the spectral components.

### Time-frequency analysis

A 10-level discrete wavelet decomposition of the signal using the discrete Meyer wavelet was calculated. The resulting decomposition is given by *W*=[*a*_10_*d*_10_*d*_9_ ⋯ *d*_1_] where *a*_10_ is the approximation coefficients and *d*_*k*_ represents detail coefficients at the *k*^*th*^-level. The signal is observed at various frequency bands thanks to this new distribution [70]. Then, the relative energy from the approximation coefficients is defined as [71]:

(15) $$ \Xi_{a}=\frac{{\|a_{10}\|}^{2}}{{\|a_{10}\|}^{2}+\sum_{k=1}^{10} {\|d_{k}\|}^{2}} (\%)  $$

(16) $$ \Xi_{d_{k}}=\frac{{\|d_{k}\|}^{2}}{{\|a_{10}\|}^{2}+\sum_{k=1}^{10} {\|d_{k}\|}^{2}} (\%)  $$

where ∥.∥ is the Euclidian norm. The relative energy which is defined by the ratio of the energy at the kth level divided by the total energy was calculated based on the wavelet transform. It denotes the distribution of energies at different frequency bands.

A wavelet entropy measures the amount of order of the signal and gives information about the distribution [71, 72]:

(17) $$ \Omega=-\Xi_{a_{10}}{log}_{2}\Xi_{a_{10}}-\sum\limits_{k=1}^{10} \Xi_{d_{k}} {log}_{2} \Xi_{d_{k}}  $$

where $\Xi _{a_{10}}$ is the relative energy. A value of *Ω* close to 0 demonstrates a concentration of wavelet energies in a fine band of levels even though a higher value of *Ω* proves an extensive band of levels (a random process).

### Statistical test

To make comparisons across sex, within tasks, types of measurements and sides in a unified manner, we fitted a series of linear mixed models with each feature as the dependent variable; sex (male/female), task (geometric/verbal/resting), measurement type (raw/envelope), side (left/right) and their full multi-way interaction as independent factors; and a subject random effect to account for multiple measurements from each participants and the resulting non-independence of observations. Next, combining both sex, to make comparisons between the levels of each of the task, measurement type and side factors within the combinations of other factors, we fit a similar mixed model but only with task, measurement type, side and their interaction as independent factors. In each case, appropriately constructed means contrasts were used to estimate the pairwise means differences of interest reported here, along with their statistical significance and 95 % confidence intervals. For cross-correlation and synchronization index features which are not side specific, we employed a largely similar strategy but omitted side from the list of independent factors. SAS version 9.3 (SAS Institute, Inc., Cary, North Carolina) was used for the mixed model analysis. MATLAB (MathWorks, Natick, MA, United States) was used for feature extraction.

## Results

Firstly, the effect of the end-tidal carbon dioxide level which does not influence features (ACA diameter) is not taken into consideration [73, 74], as we did not observe any relations between signal features and end-tidal carbon dioxide levels. Furthermore, participants did not exhibit any excessive head movements. Secondly, feature values for the raw and the envelope signals are displayed in tables in the form of (*mean*±*standarddeviation*) according to experimental conditions: the 45-seconds resting-state is indicated by a “R”, the word-generating task is indicated by a “W” and the geometric task is indicated by a “G” in the tables. Using the calculated feature values, we examined the effects of lateralization, sex, handedness and tasks on the features. A, F, M, RH and LH denote “all participants”, “female participants”, “male participants”, “right-handed participants” and “left-handed participants,” respectively.

### Time features

Table 3 presents time feature values for all participants in the raw and the envelope signals while Table 4 shows significant results in time domain concerning the handedness and sex effects in time domain.

**Table 3:** Time features from raw (denoted by the subscript r) and envelope (denoted by the subscript e) CBFV signals (* denotes multiplication by 10^−3^)

	Standard deviation		Skewness		Kurtosis		Cross-correlation
	R-ACA	L-ACA	R-ACA	L-ACA	R-ACA	L-ACA	
Rr	0.12 ± 0.05	0.12 ± 0.05	(-2.62 ± 0.15)*	(-0.59 ± 4.77)*	4.10 ± 2.06	4.43 ± 3.58	(5.67 ± 9.13)*
Wr	0.12 ± 0.05	0.12 ± 0.05	(-0.55 ± 9.02)*	0.89 ± 10.2)*	3.57 ± 0.90	4.26 ± 2.60	(6.96 ± 9.11)*
Gr	0.12 ± 0.04	0.12 ± 0.05	(-1.11 ± 9.38)*	(-1.26 ± 4.69)*	3.74 ± 0.95	4.42 ± 2.87	(6.50 ± 8.59)*
Re	15.9 ± 4.76	15.1 ± 5.98	1.17 ± 0.93	1.07 ± 0.58	5.95 ± 6.51	5.17 ± 2.52	0.89 ± 0.06
We	16.0 ± 4.72	15.2 ± 5.93	1.20 ± 0.90	1.13 ± 0.67	5.94 ± 6.16	5.50 ± 3.22	0.89 ± 0.06
Ge	15.9 ± 4.95	14.5 ± 5.87	1.03 ± 0.46	1.11 ± 0.63	4.74 ± 1.80	5.41 ± 2.79	0.90 ± 0.05

**Table 4:** Significant time features from raw (denoted by the subscript r) and envelope (denoted by the subscript e) CBFV signals where *p*<0.05

Signal	ACA	Feature	Group	Group 1	Group 2
e	R-ACA	Skewness	R	M: 1.81 ± 1.09	F: 0.91 ± 0.73
e	R-ACA	Skewness	W	M: 1.50 ± 1.13	F: 0.95 ± 0.50
e	L-ACA	Skewness	G	M: 1.40 ± 0.63	F: 0.87 ± 0.62
e	R-ACA	Kurtosis	R	M: 9.21 ± 8.01	F: 4.80 ± 3.08

No significant statistical difference was established for raw CBFV signals. On the opposite side, a few meaningful results were detected between sex on the envelope signals. Male subjects had higher skewness than female participants in R-ACA signals during resting-state (*p*=0.01) and during verbal challenge (*p*=0.02). A rise of skewness was noticed on the left ACA signals in the case of men during geometric task (*p*=0.02). Additionally, larger kurtosis was observed for men on the R-ACA during the 45-seconds resting period (*p*=0.03).

### Information-theoretic features

A summary of information-theoretic feature values and statistical differences (handedness and sex effects) in information-theoretic approach are presented in Tables 5 and 6 for the raw and the envelope signals.

**Table 5:** Information-theoretic features from raw (denoted by the subscript r) and envelope (denoted by the subscript e) CBFV signals

	LZC		Entropy rate		Index synchronization
	R-ACA	L-ACA	R-ACA	L-ACA	
Rr	0.69 ± 0.03	0.68 ± 0.04	0.30 ± 0.14	0.35 ± 0.19	0.33 ± 0.17
Wr	0.69 ± 0.02	0.68 ± 0.03	0.28 ± 0.13	0.34 ± 0.18	0.32 ± 0.16
Gr	0.69 ± 0.02	0.68 ± 0.04	0.30 ± 0.14	0.37 ± 0.19	0.34 ± 0.17
Re	0.67 ± 0.04	0.67 ± 0.03	0.06 ± 0.07	0.04 ± 0.06	0.14 ± 0.08
We	0.67 ± 0.04	0.68 ± 0.03	0.06 ± 0.07	0.04 ± 0.06	0.14 ± 0.08
Ge	0.68 ± 0.03	0.67 ± 0.03	0.05 ± 0.05	0.04 ± 0.06	0.17 ± 0.10

**Table 6:** Significant information-theoretic features from raw (denoted by the subscript r) and envelope (denoted by the subscript e) CBFV signals where *p*<0.05

Signal	ACA	Feature	Group	Group 1	Group 2
e	R-ACA	LZC	R	M: 0.65 ± 0.04	F: 0.69 ± 0.04
e	L-ACA	LZC	R	M: 0.66 ± 0.03	F: 0.70 ± 0.04
e	R-ACA	LZC	W	M: 0.65 ± 0.04	F: 0.68 ± 0.03
e	L-ACA	LZC	W	M: 0.66 ± 0.04	F: 0.69 ± 0.04

Multiple comparison test revealed significant results on LZC between sex on the envelope signals during resting periods and during word-generation challenges. Female had higher LZC in the R-ACA and the L-ACA signals during both periods (*p*<0.05).

### Frequency-domain features

Tables 7 and 8 present frequency feature values and significant results (handedness and sex effects) in the raw and the envelope signals.

**Table 7:** Frequency features from raw (denoted by the subscript r) and envelope (denoted by the subscript e) CBFV signals

	Spectral centroid		Peak frequency		Bandwidth	
	R-ACA	L-ACA	R-ACA	L-ACA	R-ACA	L-ACA
Rr	980 ± 193	939 ± 213	561 ± 308	564 ± 214	723 ± 116	564 ± 214
Wr	994 ± 184	950 ± 208	540 ± 239	584 ± 221	723 ± 116	696 ± 151
Gr	990 ± 192	939 ± 211	567 ± 269	527 ± 257	718 ± 120	690 ± 152
Re	13.3 ± 4.50	14.0 ± 4.30	0.36 ± 0.50	0.23 ± 0.46	13.5 ± 1.73	13.5 ± 1.43
We	13.5 ± 4.62	14.0 ± 4.30	0.38 ± 0.51	0.39 ± 0.55	13.5 ± 1.74	13.6 ± 1.47
Ge	13.5 ± 4.5	14.1 ± 4.36	0.45 ± 0.55	0.33 ± 0.51	13.5 ± 1.60	13.5 ± 1.37

**Table 8:** Significant frequency features from raw (denoted by the subscript r) and envelope (denoted by the subscript e) CBFV signals where *p*<0.05

Signal	ACA	Feature	Group	Group 1	Group 2
r	R-ACA	Spectral Centroid	R	M: 855 ± 198	F: 1090 ± 225
r	R-ACA	Spectral Centroid	W	M: 913 ± 204	F: 1060 ± 181
r	R-ACA	Spectral Centroid	G	M: 896 ± 203	F: 1066 ± 192
r	R-ACA	Bandwidth	R	M: 641 ± 107	F: 734 ± 118
r	R-ACA	Bandwidth	W	M: 675 ± 137	F: 763 ± 139
r	R-ACA	Bandwidth	G	M: 665 ± 141	F: 760 ± 138

Meaningful results were only noticed on sex in raw CBFV signals. The spectral centroid of raw R-ACA CBFV signals increased in the case of women during cognitive challenges and 45-seconds resting period (*p*<0.02). Moreover, the bandwidth values of R-ACA was larger from female results during rest/verbal/geometric processes (*p*<0.05).

### Time-frequency features

**Fig. 3:**
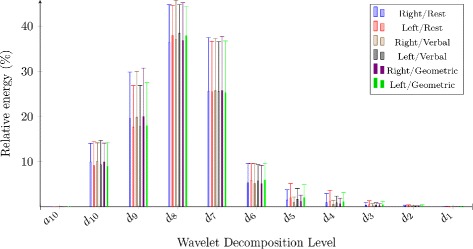
The 10th level wavelet decomposition of raw signals

**Fig. 4:**
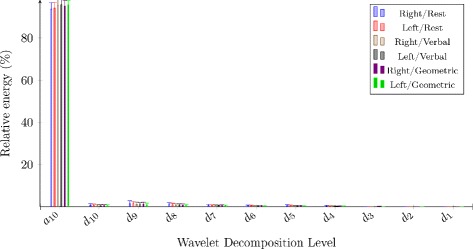
The 10th level wavelet decomposition of envelope signals

Table 9, Fig. 3 and Fig. 4 present the wavelet entropy values and the feature values of wavelet energy decomposition for raw and the envelope signals. Table 10 shows significant results for both signals (handedness and sex effects).

**Table 9:** Wavelet entropy values for raw and envelope CBFV signals

		RAW		ENVELOPE	
		R-ACA	L-ACA	R-ACA	L-ACA
Wavelet entropy	R	2.10 ± 0.23	2.12 ± 0.25	0.50 ± 0.19	0.47 ± 0.17
	W	2.07 ± 0.17	2.10 ± 0.22	0.39 ± 0.17	0.36 ± 0.16
	G	2.07 ± 0.19	2.12 ± 0.26	0.40 ± 0.18	0.35 ± 0.13

**Table 10:** Significant time-frequency features from raw (denoted by the subscript r) and envelope (denoted by the subscript e) CBFV signals where *p*<0.05

Signal	ACA	Feature	Group	Group 1	Group 2
r	R-ACA	$\Xi _{d_{10}}$	R	M: 6.92 ± 3.08	F: 11.4 ± 4.71
r	R-ACA	$\Xi _{d_{10}}$	W	M: 8.23 ± 4.05	F: 11.7 ± 3.25
r	R-ACA	$\Xi _{d_{10}}$	G	M: 7.90 ± 4.09	F: 11.7 ± 3.35
r	R-ACA	$\Xi _{d_{7}}$	R	M: 32.9 ± 12.0	F: 19.1 ± 8.02
r	L-ACA	$\Xi _{d_{7}}$	R	M: 30.3 ± 12.5	F: 21.2 ± 9.08
r	R-ACA	$\Xi _{d_{7}}$	W	M: 29.8 ± 12.2	F: 22.4 ± 9.73
r	L-ACA	$\Xi _{d_{7}}$	W	M: 29.7 ± 11.2	F: 22.1 ± 9.64
r	R-ACA	$\Xi _{d_{7}}$	G	M: 30.4 ± 12.7	F: 21.9 ± 9.97
r	L-ACA	$\Xi _{d_{7}}$	G	M: 29.5 ± 11.4	F: 21.8 ± 10.2

The multiple comparison test revealed signal information in time-frequency domain. Sex had effects on R-ACA and L-ACA raw signals. The relative energy *d*_10_ of R-ACA outcomes increased in the case of women during resting state and during mental processes (*p*<0.02), while decreasing in R-ACA and L-ACA *d*_7_ in the case of women during rest periods and cognitive tasks (*p*<0.04). For envelope results, cognitive challenges had some impact on R-ACA and L-ACA envelope signals in comparison with the baseline results, i.e. 45-seconds resting periods. A lower wavelet entropy was highlighted on both sides of ACA during cognitive processes (*p*<0.04).

On the other hand, 94 % of energy were concentrated around the approximation band *a*_10_ for the envelope signals. Therefore, for these signals, we only considered the *a*_10_ level. Statistical differences in R-ACA and L-ACA *a*_10_ were noticed: the mental periods showed larger *a*_10_ than rest periods (*p*<0.02).

### Comparison between raw and envelope signals

Results demonstrated differences between raw signals and envelope signals and showed low *p*-values except for results in Table 11.

**Table 11:** Absence of statistical difference between raw and envelope CBFV signals where *p*>0.06

Multiple	Rest periods		Verbal tasks		Geometric tasks	
	R-ACA	L-ACA	R-ACA	L-ACA	R-ACA	L-ACA
Kurtosis	A, F	A, M, F	F	A, M, F	A, M, F	A, M, F
LZC	F	A, M, F	F	A, M, F	A, M, F	A, M, F
$\Xi _{d_{5}}$	M, F	M	A, M, F	M	A, M, F	M
$\Xi _{d_{4}}$	A		A		A	
$\Xi _{d_{2}}$	M		M	M	M	
$\Xi _{d_{1}}$	A, M, F	A, M, F	A, M, F	A, M, F	A, M, F	M, F

## Discussion

Raw and envelope signals were significantly different as demonstrated by the features describing their probability density functions. No effect of handedness was found on time domain features while the sex effects were exhibited on the R-ACA and L-ACA raw and envelope signals. Higher kurtosis proved that the CBFV variations are grouped around one value [75], while larger skewness highlighted higher signal assymetry [53]. R-ACA envelope signals from female participants seemed more dispersive and more symmetrical than from male subjects during the rest and verbal periods. Hence, the cerebral blood flow changed with a wider range in females than in males during resting-state and word-generation processes on the right side of ACA.

Time-domain outcomes did not reveal differences between signal characteristics during resting-state periods and during mental challenges. For example, the cross-correlation value was close to zero for raw signals implying low signal dependence in the time domain. Previous studies highlighted the dependence of signals between the two sides of MCA and the evolution of blood flow velocity during cognitive processes. Lateralization was introduced during the geometric task and the word-generation task. It was shown that there was a hemispheric lateralization due to an increase of the cerebral blood flow velocity during cognitive tasks [32, 33, 35]. The geometric task led to a dominance of R-MCA while the verbal task results in a dominance of L-MCA. Moreover, the cerebral blood flow in MCA is closely linked to the flow in ACA. Thus, an identical hemispheric dominance should be identified using results from ACA. Changes in the ACA blood flow and the possible lateralization observed in the flow can be explained by the changes in the MCA blood flow while performing cognitive processes. Nonetheless, time domain results from the current ACA study did not confirm cerebral lateralization during mental challenges.

Raw signals and envelope signals showed distinct cerebral blood flow characteristics which demonstrated the significance of extraction of envelope signals and preservation of raw signals from a statistical point of view. The envelope signals also had higher standard deviation and skewness than the raw signals. Raw signals centralized information around a value because of lower skewness and standard deviation. However, envelope signals exhibited higher cross-correlation values (*CC*>0.89) than raw signals which proved that there was a low dependence on raw signals in the time domain.

When considering the information theoretic domain, sex effects were observed on randomness and complexity of the envelope signals. In fact, the Lempel-Ziv complexity exhibited differences between men and women. Right-sided and left-sided envelope signals from women were more complex than signals from men during resting-state period and word-generation processes. Rest periods and verbal tasks implied that the left and right ACA blood flow speed was distinguishable between men and women. Indeed, the envelope signals vary with higher fluctuations in the case of women.

In the frequency domain, raw signals exhibited higher frequency characteristics than envelope signals. As a matter of fact, envelope signals revealed a low-pass structure, while the raw signals presented a band-pass structure. When differentiating sex effects, spectral centroid frequencies and bandwidth of R-ACA raw signals from female participants had higher values than those from men during the geometric task. This finding implied an increase of right-sided blood flow above baseline was more pronounced for women than for men during geometric tasks. Sex differences during mental stimuli have been widely studied, particularly during spatial challenges. Previous publications underlined distinct hemispheric dominance seen in women and in men [40, 76, 77]. Women revealed activation of other right brain regions during geometric tasks [76]. It could be explained by two distinct strategies of task solving/the anatomical brain structure/sex hormones [78–81]. A rise of centroid frequency was also noticed during the resting-state and verbal tasks. It appeared that female subjects showed a higher right-sided CBFV baseline. These distinct baseline metabolisms may be caused by emotional brain responses (i.e. sex hormones) or by anatomical brain differences [79–82]. The frequency domain analysis did not indicate the existence of handedness-based difference.

From the time-frequency point of view, lower wavelet entropy values highlighted that right-sided and left-sided envelope signals were more ordered during cognitive periods than during 45-second resting-state periods. In addition, the rise of wavelet energy *a*_10_ proved that cognitive tasks led to modifications of CBFV signals comparing to resting state. Time-frequency outcomes did not expose major changes into brain functioning during mental tasks. We did not observe neither handedness nor sex effects on *a*_10_ values for R-ACA and L-ACA raw and envelope signals.

The uneven small number of subjects in each group may also be important limitation in the outcomes about the sex effects (i.e. 20 subjects) and handedness effects on brain response (i.e. 16 right-handed and 3 left-handed subjects). Therefore, the results obtained after analyzing comparisons between right and left-handed subjects and between male and female participants are not as pronounced. Given the low number of ambidextrous participants (i.e. 1 ambidextrous subject), we examined the significant *p*-values between right and left-handed participants.

## Conclusions

In this study, the evolution of the cerebral blood flow velocities in left and right ACA was investigated during three different tasks: a mental rotation task, a word generation task and resting periods between cognitive tasks. Characteristics of the raw signals and the envelope signals were analyzed in time, frequency, and time-frequency domains. Significant results have been obtained through the use of both classic/modern methods (i.e. envelope/raw, time and frequency/information-theoretic and time-frequency domains). The time and the information-theoretic results underlined modifications of shape distribution and randomness. The acquired data in the frequency domain presented a low-pass characteristic in the case of envelope signals while the raw signals presented band-pass characteristics. In the time-frequency analysis, the distribution of wavelet energies for the envelope signals was around the low frequencies during cognitive activities. Finally, differences were obtained for the raw and envelope signals based on sex effects. Distinct hemispheric functioning between men and women was highlighted during each process. A few significant statistical differences demonstrated the different brain response.
